# Distinct bacterial community structure and composition along different cowpea producing ecoregions in Northeastern Brazil

**DOI:** 10.1038/s41598-020-80840-x

**Published:** 2021-01-12

**Authors:** Luciana de Sousa Lopes, Lucas William Mendes, Jadson Emanuel Lopes Antunes, Louise Melo de Souza Oliveira, Vania Maria Maciel Melo, Arthur Prudêncio de Araujo Pereira, Antonio Félix da Costa, José de Paula Oliveira, Cosme Rafael Martínez, Marcia do Vale Barreto Figueiredo, Ademir Sérgio Ferreira Araujo

**Affiliations:** 1 Soil Quality Lab, Agricultural Science Center, Federal University of Piauí, Teresina, PI Brazil; 2 Center for Nuclear Energy in Agriculture, University of Sao Paulo, Piracicaba, SP Brazil; 3 Laboratório de Ecologia Microbiana e Biotecnologia, Federal University of Ceará, Fortaleza, CE Brazil; 4 Soil Science Department, Federal University of Ceará, Fortaleza, CE Brazil; 5 Instituto Agronômico de Pernambuco, Recife, PE Brazil; 6 Centro de Ciências Exatas e da Natureza, Universidade Federal da Paraíba, João Pessoa, PB Brazil

**Keywords:** Microbial ecology, Molecular ecology, Tropical ecology

## Abstract

Soil microbial communities represent the largest biodiversity on Earth, holding an important role in promoting plant growth and productivity. However, the knowledge about how soil factors modulate the bacteria community structure and distribution in tropical regions remain poorly understood, mainly in different cowpea producing ecoregions belonging to Northeastern Brazil. This study addressed the bacterial community along three different ecoregions (Mata, Sertão, and Agreste) through the16S rRNA gene sequencing. The results showed that soil factors, such as Al^3+^, sand, Na^+^, cation exchange excel, and total organic C, influenced the bacterial community and could be a predictor of the distinct performance of cowpea production. Also, the bacterial community changed between different ecoregions, and some keystone groups related to plant-growth promotion, such as *Bradyrhizobium,* Bacillales, Rhizobiales, and *Solibacillus,* were correlated to cowpea yield, so revealing that the soil microbiome has a primordial role in plant productivity. Here, we provide evidence that bacterial groups related to nutrient cycling can help us to increase cowpea efficiency and we suggest that a better microbiome knowledge can contribute to improving the agricultural performance.

## Introduction

Soil is recognized as a functional environment, essential to the stability of the terrestrial ecosystem, being composed of a large microbial diversity^1,2^. In this environment, bacteria are the most abundant microbial groups and play fundamental roles, especially on nutrient cycling and, consequently, contributing to plant growth-promotion^3–6^. Thus, these microbial communities are important to sustain ecosystem services and crop productivity^7,8^.

In soil, the structure of the bacterial community is influenced by chemical and climatic drivers, especially in tropical regions where the soil fertility, temperature, and rainfall present high variation^9^. However, recent studies have reported the soil factors as the most significant drivers of the bacterial community’s structure, contributing to their functions and spatial distribution^5,9–11^. For instance, Xue et al.^5^ assessed the bacterial community across a latitude gradient in Australia and found that soil factors (especially soil nutrients) contributed mostly to the spatial abundance of the community than environmental conditions (e.g., temperature, elevation) or agricultural practices.

The Pernambuco State, located at Northeastern Brazil, presents three distinct ecoregions known as Mata, Agreste, and Sertão, with contrasting soil and climate conditions. In these regions, the most variable conditions are soil pH, fertility (macro and micronutrients, and organic matter), temperature, and annual rainfall^12^. Regarding the climate (temperature and rainfall), Mata presents a humid tropical climate, an average temperature of 28 °C, and regular rainfall; while Sertão presents a semiarid climate, an average of temperature of 32 °C, scarce and poorly distributed rainfall. Agreste is the transition region between Mata and Sertão and presents an average of temperature of 25 °C, and characteristics of both humid and semiarid climate^13^. Particularly, these regions are important to produce cowpea (*Vigna unguiculata*), a special type of legume species used as a protein source to the local population, and the contrasting soil conditions found in these regions have promoted different performance of cowpea yield^14,15^.

Therefore, the differences in cowpea yield found in these regions have been attributed to soil chemical factors. Although is known that soil factors present a strong influence on plant performance, we argue that the soil bacterial community may also contribute to cowpea yield due to the role of the microbes in the soil nutrient cycle. Thus, it is necessary to disentangle the microbial communities in these soils and evaluate the effect of the soil factors on their composition, structure, and diversity. A better understanding of the major drivers that influence these communities could contribute to improving the cowpea performance and, at the same time, identify the most important microbial groups in these soils.

In this work, we assessed how the bacterial community varied along the three distinct ecoregions found in Northeastern Brazil. We hypothesized that (1) differences in soil chemical properties and environmental factors select distinct groups of bacteria; and (2) differences in cowpea performance can be attributed to differences in bacterial groups. To address these points, we used high-throughput 16S rRNA gene sequencing in soil samples from six different locations belonging to the three ecoregions found in the state of Pernambuco, Brazil.

## Results

The analysis of the soil properties revealed a great variability of the attributes among the areas (Table 1). In general, the site ‘Surubim’ (AS) from the Agreste region presented higher values for most of the measured parameters, such as P, Ca^2+^, Mg^2+^, K^+^, the sum of basis (SB), and cation exchange capacity (CEC). The region of Mata presented a higher total organic C and sand proportion than other regions. The Agreste area presented higher values of Al^3+^ and Na^+^. The temperature was markedly different between regions, with Sertão presenting the highest temperatures while Agreste presented the lowest.

**Table 1:** Soil properties, temperature, altitude, and cowpea yield of the evaluated location.

	Mata		Sertão		Ageste	
	VSA	ZMI	BSF	SAA	AS	AL
Soil pH	6.4 a	5.2 b	6.6 a	4.5 c	6.5 a	6.5 a
P (mg kg^−1^)	25 c	13 d	36 c	3.0 e	175 a	125 b
Ca^2+^ (cmol_c_ kg^-1^)	2.6 b	0.7 d	2.4 b	0.3 d	4.5 a	2.1 bc
Mg^2+^ (cmol_c_ kg^−1^)	1.3 b	0.4 d	0.8 c	0.4 d	2.0 a	0.9 c
Al^3+^ (cmol_c_ kg^−1^)	0 b	0.1 b	0 b	0.5 a	0 b	0 b
Na^+^ (cmol_c_ kg^−1^)	0.11 b	0.04 d	0.17 a	0.02 d	0.07 c	0.04 cd
K^+^ (cmol_c_ kg^−1^)	0.28 b	0.02 c	0.19 b	0.03 c	1.40 a	0.37 b
SB (cmol_c_ kg^−1^)	4.3 b	1.3 d	3.7 b	0.9 d	7.7 a	2.5 c
CEC (cmol_c_ kg^-1^)	6.4 b	3.5 d	4.5 c	4.5 c	9.7 a	4.5 c
V (%)	68 b	34 c	81 a	21 d	80 a	66 b
m (%)	0 c	8.3 b	0 c	62.1 a	0 c	0 c
TOC (g kg^−1^)	11 a	7.5 b	3.2 c	4.8 c	4 c	8.6 b
Sand (%)	90 a	89 a	75 b	80 b	68 c	63 c
Silt (%)	2 b	4 b	15 a	1.5 b	16 a	15 a
Clay (%)	8 d	7 d	9 cd	19 a	15 b	12 c
Soil class^a^	Ultisol	Ultisol	Entisol	Oxisol	Alfisol	Alfisol
Temperature (^°^C)	27.5 b	27 b	30 a	31 a	25 c	25 c
Altitude (m)	160	13	305	622	414	661
Yield (kg ha^−1^)	1094 a	511 b	330 e	286 c	245 d	426 f.

^a^Soil Taxonomy; *SB* sum of basis, *CEC* cation exchange capacity, V (%): basis saturation, m (%) Al^3+^ saturation, *TOC* total organic carbon, *VSA* Vitoria de Santo Antão, ZMI, Itapirema; AS, Surubim; AL, Lajedo; *BSF* Belém do São Francisco, SAA, Araripina.

**Figure 1:**
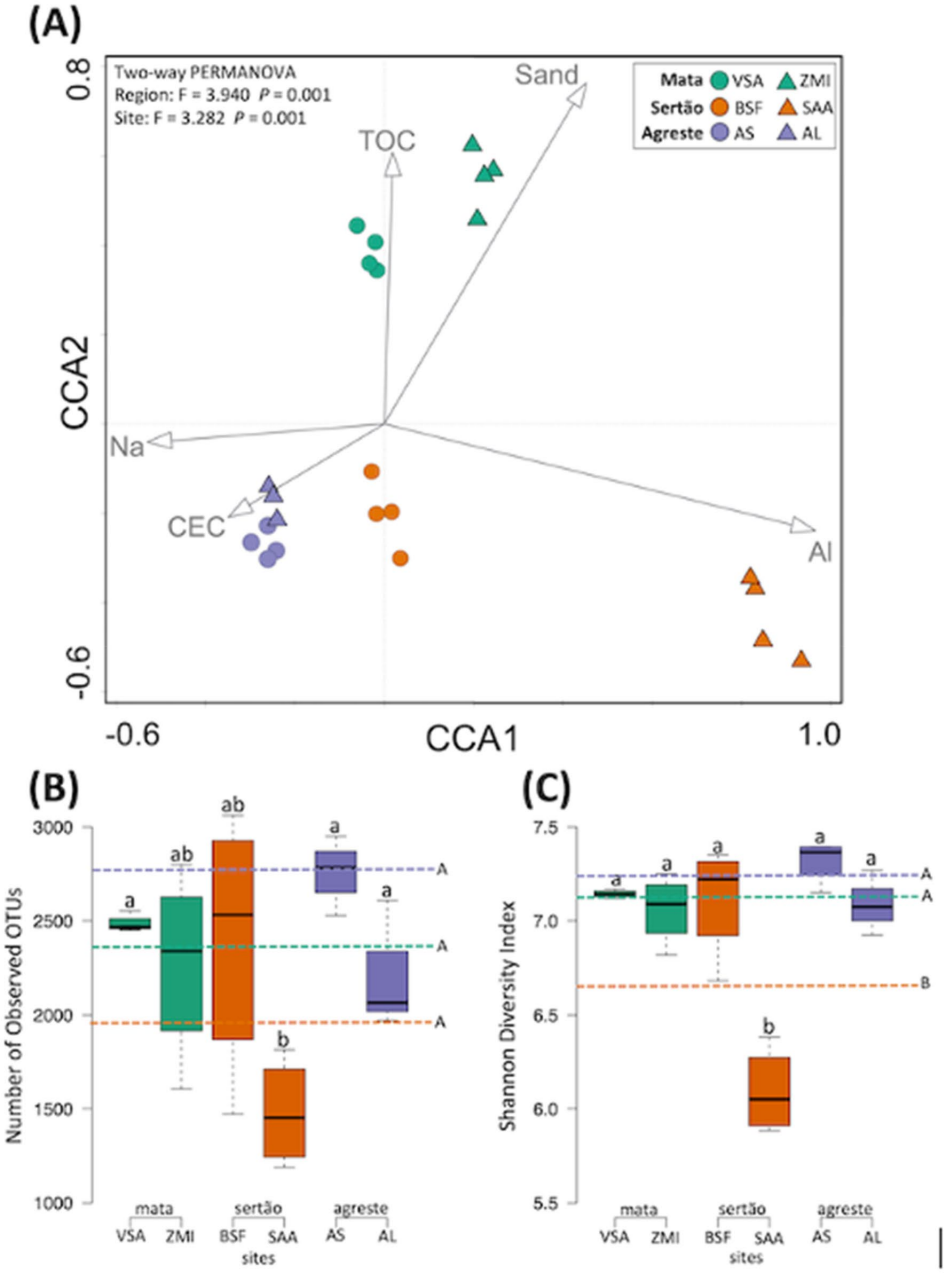
Structure and diversity of the bacterial community in soils along three distinct ecoregions under cowpea cultivation based on the 16S rRNA gene. (**A**) Canonical correspondence analysis of the bacterial community patterns and environmental parameters constructed in Canoco 4.5. Arrows indicate correlation between environmental parameters and microbial profile. Only significant environmental factor is shown. The analysis of permutation (PERMANOVA) is indicated in the upper left corner of the graph. (**B**) Richness and (**C**) Diversity measurements of the bacterial communities at OTU level. Error bars represent the standard deviation of four independent replicates. The dashed lines represent the average value for each region. Different lower-case letters refer to significant differences between each site and different upper-case letters refer to significant differences between regions. The comparison is based on Tukey’s HSD test using the PAST software (*p* < 0.05).

The canonical correspondence analysis (CCA) was used to evaluate the structure of the bacterial communities and their relationship with the soil properties (Fig. 1A). This analysis clustered the samples according to the regions (PERMANOVA F = 3.940, *p* = 0.001), and sites (PERMANOVA F = 3.282, *p* = 0.001). The explanatory variables accounted for 39.78% of the total variation. Furthermore, according Forward Selection analysis followed by Monte Carlo test the general bacterial community structure correlated with Al^3+^ (F = 2.8; *p* = 0.001), sand (F = 2.3; *p* = 0.001), Na^+^ (F = 1.9; *p* = 0.001), CEC (F = 1.6; *p* = 0.001), and total organic C (F = 1.7, *p* = 0.001). More specifically, the bacterial community in Agreste correlated more to CEC and Total organic C, while the community in Sertão correlated to sand and Na^+^. On the other hand, Mata showed a positive correlation with clay content. The bacterial richness and diversity did not vary among the sites, except to soil from ‘Araripina’ (SAA) that presented the lowest bacterial richness and diversity (Fig. 1B, C). When we considered the average of bacterial diversity in each region, the Sertão presented lower diversity compared to Mata and Agreste.

**Figure 2:**
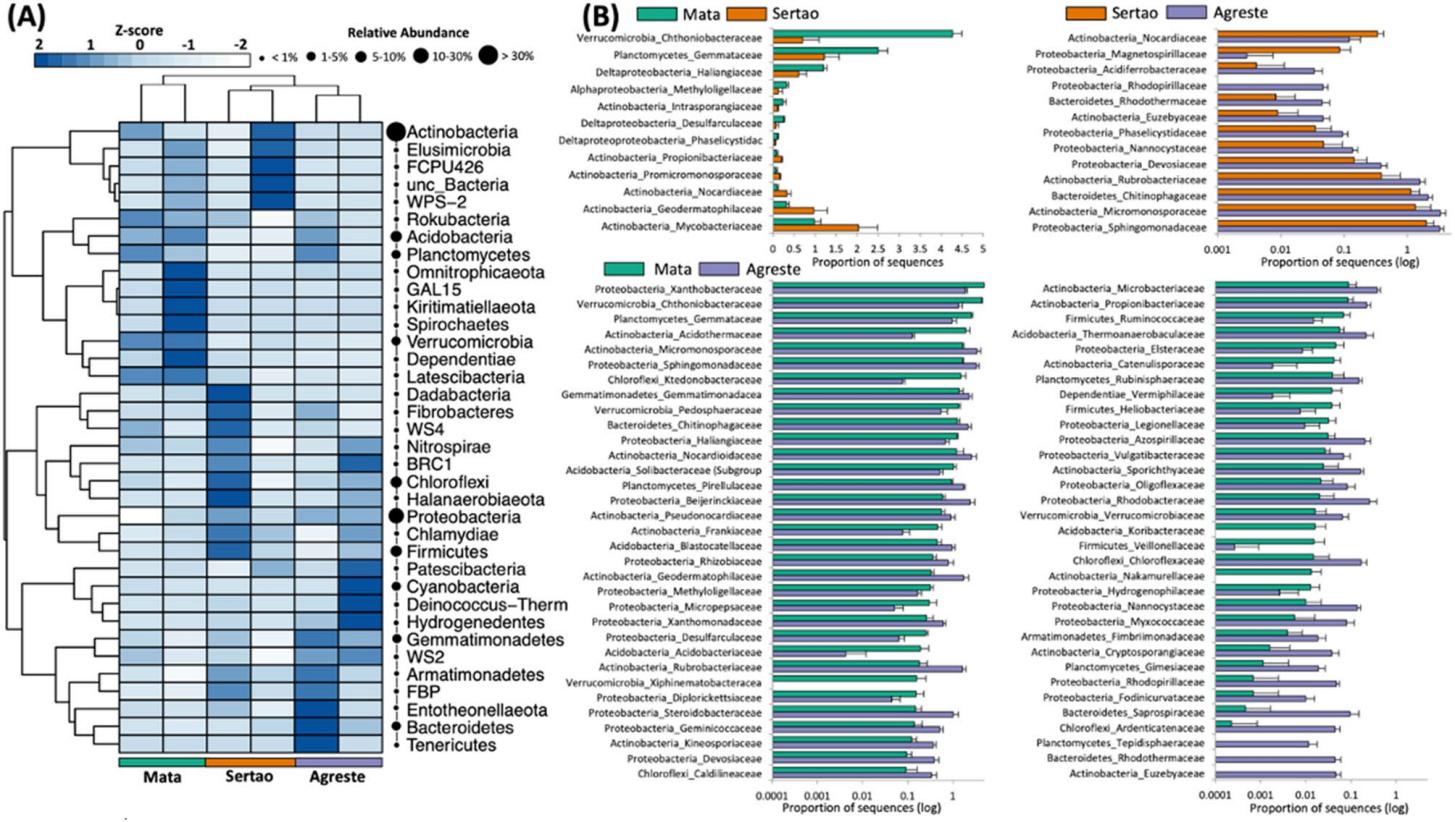
Bacterial composition in soils along three distinct ecoregions under cowpea cultivation based on the 16S rRNA gene. (**A**) Heatmap showing the differential abundance of phyla among the sites. The color key relates the heat map colors to the standard score (z-score), i.e. the deviation from row mean in units of standard deviation above or below the mean. The circles are proportional to the relative abundance of each phylum in all samples. (**B**) Scatter-plot showing the differential abundance of family among regions based on Welch’s t-test with Benjamini–Hochberg correction constructed in STAMP (*P* < 0.05).

The bacterial community was composed of 41 phyla, with the most abundant belonging to Actinobacteria (33% of the total sequences), followed by Proteobacteria (25%), Acidobacteria (9.5%), Firmicutes (8%), Chloroflexi (6.5%), Planctomycetes (4.5%), Verrucomicrobia (3.5%), Bacteroidetes (2.2%), Gemmatimonadetes (1.7%), and Cyanobacteria (1.4%), which together represented > 96% of the bacterial community (Fig. 2A). Interestingly, each region presented a dominance of different phyla. The region of Mata presented a higher abundance of the phyla Verrucomicrobia, Latescibacteria, Gal15, Rokubacteria, and Dependentia (*p* < 0.05); the region of Agreste presented a high abundance of Bacteroidetes, Gemmatimonadetes, WS2, Hydrogenedentes, and Entotheonellaeota (*p* < 0.05); and the Sertão presented a high abundance of Firmicutes, and a decrease in Acidobacteria (*p* < 0.05) when compared to the other regions (Fig. 2A).

At the family level, specific microbial groups were enriched in each region (Fig. 2B). In the comparison between Mata and Sertão, Chthoniobacteraceae (Verrumicrobia) and Gemmataceae (Planctomycetes) were abundant in Mata, and Geomdermatophilaceae and Mycobacteriaceae (Actinobacteria) were abundant in Sertão. When compared Sertão and Agreste, Nocardiaceae (Actinobacteria), and Magnetospirillaceae (Proteobacteria) were abundant in Sertão, while Rhodospirillaceae (Proteobacteria), Rhodothermaceae (Bacteroidetes), and Euzebyaceae (Actinobacteria) were abundant in Agreste. The comparison between Mata and Agreste showed the highest number of microbial groups, being Acidothermaceae (Actinobacteria), Ktenodobacteraceae (Chlroflexi), Acidobacteraceae (Acidobacteria), and Xiphinematobacteraceae (Verrumicrobia) abundant in Mata, while Euzebyaceae (Actinobacteria), Rhodothermaceae (Bacteroidetes), Tepidisphaeraceae (Planctomycetes), and Ardenticatenaceae (Chloroflexi) were abundant in Agreste.

**Figure 3:**
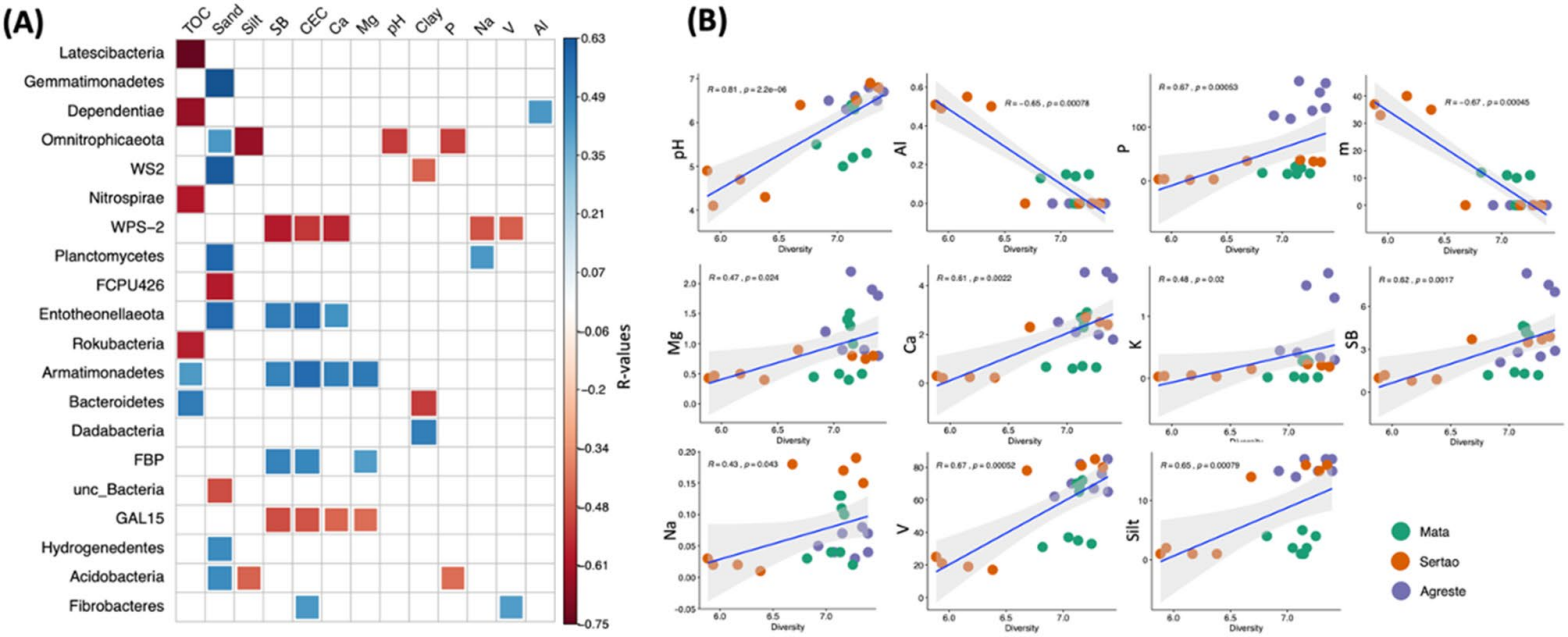
(**A**) Heatmap showing the Spearman’s rank correlation coefficients and statistical significance between phyla abundance and soil chemical parameters. Blue and red colors indicate significant positive and negative correlations, respectively (*P* < 0.05). (**B**) Spearman correlation between bacterial diversity and soil chemical parameters (*P* < 0.05). SB: sum of basis, V (%): basis saturation, m (%) Al^3+^ saturation. Heatmap constructed using the R package ‘corrplot’.

In order to analyze the correlation between individual bacterial phyla and soil physicochemical properties, we calculated all possible Spearman’s rank correlation to better show the drivers that influence the microbial community structure (Fig. 3A). The soil factors that correlated with most bacterial phyla were sand (9 phyla in total), followed by total organic C (6), CEC (6), and SB (5). For sand content, the majority of microbial phyla correlated positively. When considering S and CEC, the phyla WPS-2 and GAL15 correlated negatively, while Entotheonellaeota, Armatimonadetes, Euryarchaeota, and FBP correlated positively. The phyla that correlated with the highest number of soil properties were Armatimonadetes and WPS-2 (5 factors in total), followed by Omnitrophicaeota, Entotheonellaeota, and GAL15 (4). Further, we investigated how the soil properties influenced the bacterial diversity in these soils (Fig. 3B). The results also showed that the majority of soil drivers presented a positive correlation with microbial diversity, with the exceptions of Al^3+^ and m% (Al^3+^ saturation) that presented a negative correlation with microbial diversity (*p* < 0.05).

**Figure 4:**
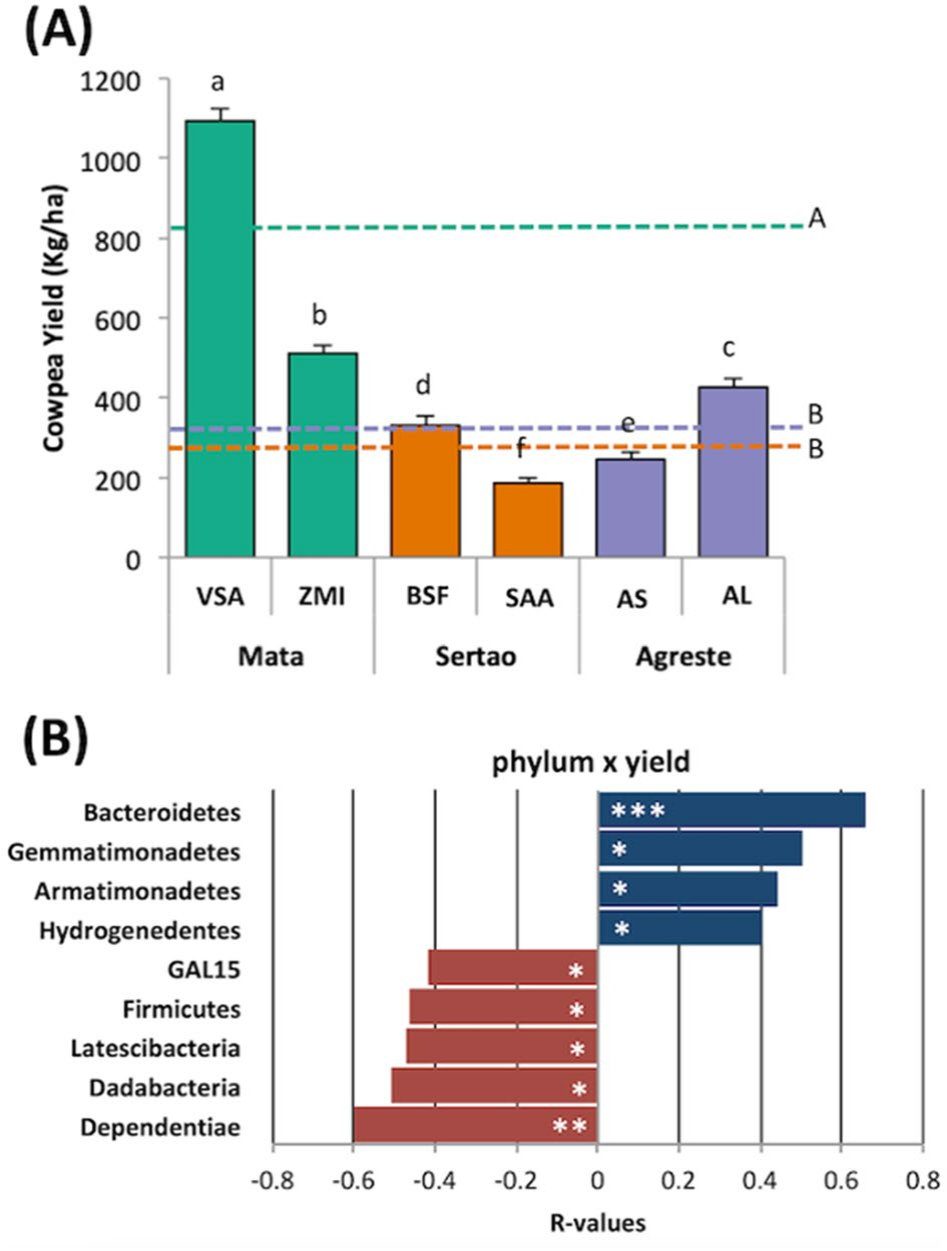
(**A**) Comparison of the cowpea yield between sites. Error bars represent the standard deviation of four independent replicates. The dashed lines represent the average value for each region. Different lower-case letters refer to significant differences between each site and different upper-case letters refer to significant differences between regions. The comparison is based on Tukey’s HSD test using the PAST software (*P* < 0.05). (**B**) Correlation between cowpea yield and bacterial phyla. Blue and red bars represent positive and negative correlation, respectively. The correlation is based on Spearman’s rank coefficient (***P < 0.001, **P < 0.01, *P < 0.05).

We further correlated the bacterial phyla with the cowpea yield in each site (Fig. 4). Interestingly, although the soil properties were very variable among the sites and regions, the cowpea productivity in the areas of Mata presented higher yield compared to other regions (Fig. 4A). The correlations analysis showed that the phyla Bacteroidetes, Gemmatimonadetes, Armatimonadetes, and Hydrogenedentes correlated positively with cowpea yield, while Dependentiae, Dadabacteria, Latescibacteria, Firmicutes, and Gal15 correlated negatively (Fig. 4B).

**Figure 5:**
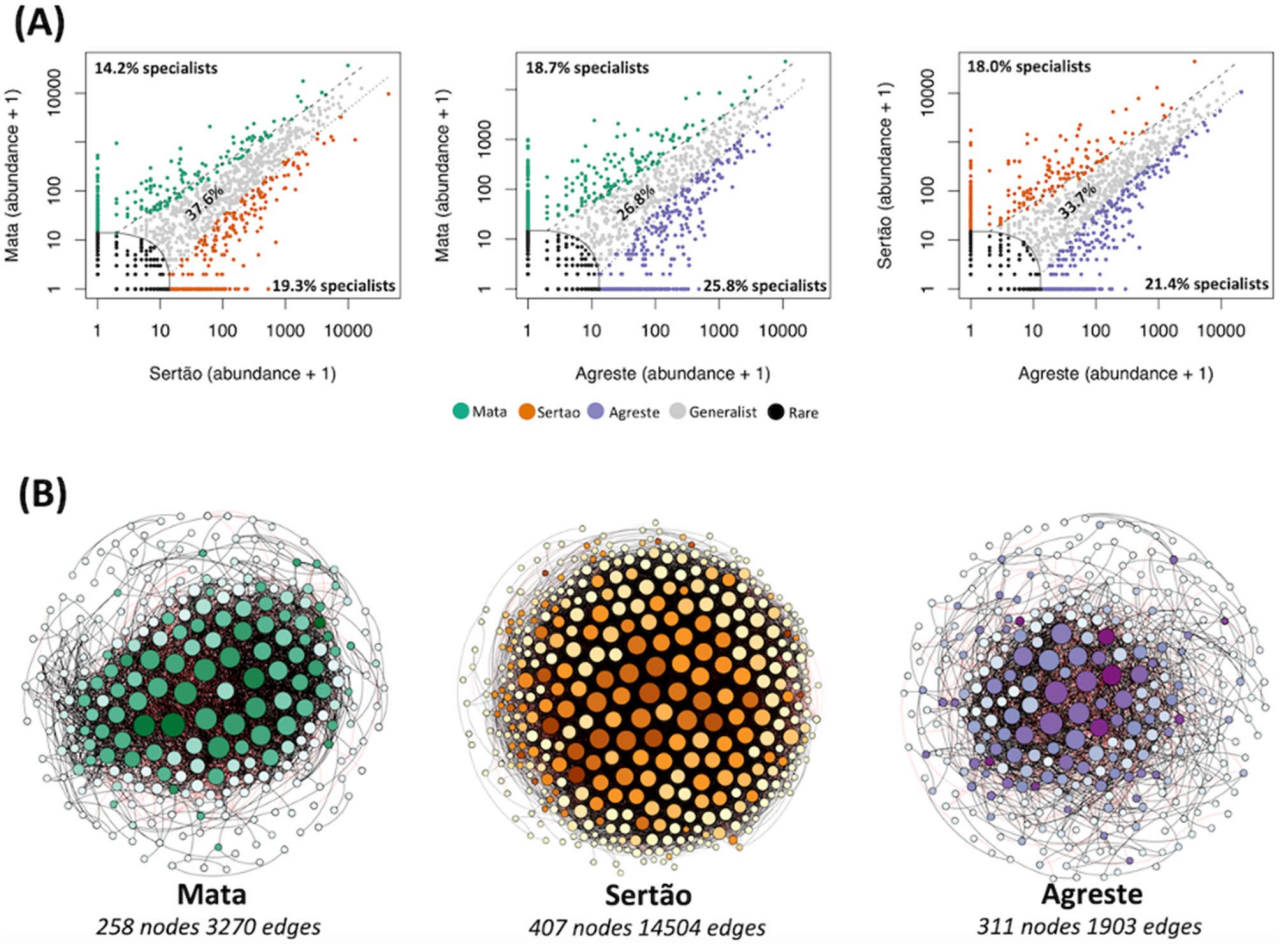
(**A**) Multinomial species classification method (CLAM) for the niche occupancy test based on pairwise comparison. The generalists (gray), specialists (green, orange, and purple), and rare (black) are indicated with their respective percentages. (**B**) Network co-occurrence analysis of the bacterial communities in soils along three distinct ecoregions under cowpea cultivation based on the 16S rRNA gene. A connection stands for SparCC correlation with magnitude > 0.9 (positive correlation–blue edges) or <  − 0.9 (negative correlation–red edges) and statistically significant (*P* ≤ 0.01). Each node represents taxa at OTU level and the size of node is proportional to the number of connections (that is, degree). The color of the nodes is based on the betweeness centrality, where darker colors indicated higher values. The network construction was made using Gephi software.

The analysis of niche occupancy revealed that the proportion of specialists and generalists varied between regions (Fig. 5A). In general, the proportion of generalists is higher than specialists in all regions. Comparing Mata with Sertão, 37.6% of the bacterial species were classified as generalists (mutually present in both sites), while 19.3% were classified as specialists in Sertão against 14.2% in the Mata region. The comparison between Mata and Agreste also showed the same pattern of a lower proportion of specialists in Mata (18.7% against 25.8% in the Agreste). Lastly, Agreste (21.4%) showed a higher proportion of specialists as compared to Sertão (18%).

Then, co-occurrence network analysis was used to compare the complexity of connections in the microbial community in each region (Fig. 5B and Table 2). Different regions presented different network compositional and topological features. In general, the result showed that Sertão exhibited more complexity (number of nodes = 407, edges = 14,504, average degree = 71.27), followed by the Mata region (number of nodes = 258, edges = 3270, average degree = 25.34), while Agreste showed the lowest complexity (number of nodes = 311, edges = 1903, average degree = 12.23). Further, we identified the keystone species in each network based on the betweenness centrality (Supplementary Table 1), defined as the number of times a node plays a role as a connector between two other nodes, considered an important ecological and biological feature within a network. Our analysis revealed that there is a complete change in the key species in each region. In Mata, the top five key species belong to the phyla Chloroflexi (Anaerolineae and Reseiflexacae), Firmicutes (Bacillales), and Proteobacteria (*Bradyrhizobium*). In Sertão, most of the keynodes belong to the phylum Actinobacteria (*Solirubrobacter* and *Rubrobacter*). In Agreste, the top five keynodes were affiliated to Actinobacteria (*Nocardioides*), Proteobacteria (Rhizobiales and Nitrosomonadaceae), Gemmatinomadetes, and Firmicutes (*Solibacillus*).

**Table 2:** Correlations and topological properties of microbiome networks from soil of Mata, Sertão, and Agreste.

Network properties	Mata	Sertão	Agreste
Number of nodes^a^	258	407	311
Number of edges^b^	3270	14,504	1903
Positive edges^c^	2119	8760	1213
Negative edges^d^	1151	5744	690
Modularity^e^	1.628	2.371	1.867
Number of communities^f^	25	21	45
Network diameter^g^	7	9	12
Average path length^h^	2.568	2.358	3.70
Average degree^i^	25.34	71.27	12.23
Av. clustering coefficient^j^	0.683	0.726	0.562

^a^Microbial taxon (at genus level) with at least one significant (*P* < 0.01) and strong (SparCC > 0.7 or < −0.7) correlation;

^b^Number of connections/correlations obtained by SparCC analysis;

^c^SparCC positive correlation (> 0.7 with *P* < 0.01);

^d^SparCC negative correlation (< −0.7 with *P* < 0.01);

^e^The capability of the nodes to form highly connected communities, that is, a structure with high density of between nodes connections (inferred by Gephi);

^f^A community is defined as a group of nodes densely connected internally (Gephi);

^g^The longest distance between nodes in the network, measured in number of edges (Gephi);

^h^Average network distance between all pair of nodes or the average length off all edges in the network (Gephi);

^i^The average number of connections per node in the network, that is, the node connectivity (Gephi);

^j^How nodes are embedded in their neighborhood and the degree to which they tend to cluster together (Gephi).

## Discussion

Here, we analyzed the bacterial community using high-throughput sequencing of the 16S rRNA gene in soils belonging to three ecoregions (comprising six different localities with cowpea production) from the state of Pernambuco, Brazil. In this study, chemical and physical properties correlated to bacterial community and showed a tiny separation between regions. Therefore, this result confirms that the different soil conditions found in these regions contributed to the distribution of bacterial communities according to distinct drivers. In Agreste, CEC and total organic C content presented a positive correlation with the bacterial community showing that high soil fertility and organic C contribute to shaping microbial communities. Indeed, it is widely known the positive influence of total organic C on the bacterial community as a source of energy and nutrients to microbial metabolism^16,17^. Also, in Agreste, there is a predominance of Alfisol soil, which presents high natural fertility^18^. However, the cowpea performance in Agreste is low and, thus, further studies can be necessary to address the functionality of these bacterial groups in Agreste that, although can be positively influenced by soil fertility, do not influence positively the cowpea production. In Sertão, the bacterial community correlated with sand and Na^+^, suggesting that different bacterial groups are more adapted to unfavorable conditions, such as high sandy and salinized soils. In this region, the average of cowpea production is low and reflects the soil conditions found in Sertão. The bacterial community in soils from Zona da Mata was correlated significantly with clay, showing that the soil particle is an important driver of the bacterial community and also contributes to the increase of soil fertility and plant growth^19,20^. Indeed, the highest cowpea production was found in the localities from Mata. To further understand the effect of the soil microbiome on the cowpea production, we correlated bacterial phyla with yield and showed that Bacteroidetes presented the most significant positive correlation. Some studies suggested that representatives of this phylum could affect plant growth and health^21^. For example, the genera *Flavobacterium* and *Chryseobacterium* have been associated with plant growth promotion and disease protection^22,23^.

The microbial richness (i.e. the number of observed operational taxonomic units) and the Shannon diversity index do not change between areas but showed a strong reduction in Sertão, particularly in SAA. SAA presents a predominance of Oxisol soils that are considered the most weathered soil class, showing low nutrient content and soil pH^24^. Thus, SAA showed lower soil pH and, more importantly, lower P content as compared to others. The soil pH has been shown to be generally correlated with microbial diversity, which may be a result of the integration of the pH with other soil properties^25^. Also, phosphorus is an essential nutrient to microbial metabolism, being part of the important microbial macromolecules, such as DNA, RNA, ATP, and its lower availability can limit both microbial and plant life^26^. Altogether, unfavorable pH and low P availability may be responsible for lower quality and fertility of the soil in this region^27^.

In line with the hypothesis (1), we found distinct groups of bacteria that were selected by the soil conditions found in each region. Sertão presented a high abundance of Actinobacteria and particularly SAA showed more than 30% of relative abundance of this phylum. It suggests that such soil conditions found in Sertão contributed to this high abundance of Actinobacteria since they are diverse and adapted to extreme conditions, and it includes bacterial groups known as acidtolerant, alkalitolerant, psychrotolerant, thermotolerant, halotolerant, and haloalkalitolerant or xerophiles mechanisms^28^. Sertão presents lower rainfall, high soil temperature, and nutrient-poor soils, characteristics that contribute to Actinobacteria establishment. It also may explain the significant reduction in alpha diversity metrics and partially explain the fact that we found a more connected microbial community (networks with more nodes and edges) in SAA. Also, under lower soil diversity index the plant can present deficient recruitment of bulk soil organisms to their rhizosphere system^29^. This represents a critical disbalance in the cowpea production area since the bulk soil diversity plays an important role in the root nodule microbiome establishment^30^, a crucial compartment to the nitrogen biological fixation process. Agreste presented a higher abundance of Bacteroidetes, a phylum that includes plant-growth promoting bacteria and cellulose decomposing bacteria^31^. However, the cowpea production in this area presented intermediate values compared to Mata and Sertão. The family Rhodothermaceae was one of the most abundant bacterial group found in this area, and they are halo-thermophile with cellulolytic and xylanolytic activities^32^. On the other hand, Mata presented a higher abundance of Verrucomicrobia and Rokubacteria compared to the other regions. The phylum Verrucomicrobia is extremely sensitive to changes in chemical factors linked to soil fertility, with a significant decrease after the conversion of native forest to agriculture^33^. This phylum is also linked to soil moisture^34^, which could explain their higher abundance in Mata since these areas present higher rainfall and soil moisture. The new proposed bacterial phylum Rokubacteria was found in high abundance in Brazilian rainforest soils, with the potential to oxidize methane^35^. Considering that our areas of Mata have similar characteristics of other Brazilian rainforests, this could explain their higher abundance in these areas.

The Spearman correlation showed positives interactions between bacterial community and soil properties, such as pH, P, Mg^2+^, Ca^2+^, K^+^, and SB, indicating the important link between bacterial community and soil fertility. Particularly, SB, CEC, Ca^2+^, and Mg^2+^ showed a strong positive correlation, mainly with Entotheonellaeota and Armatimonadetes. Our analysis also showed that specific soil properties correlated with bacterial diversity, with most of them having a positive correlation. This result highlights the link between bacterial diversity and soil fertility, as shown by the higher cowpea production in the areas with higher bacterial diversity. It is well known that biodiversity enhances ecosystem stability and productivity, where higher microbial diversity promotes soil ecosystem functioning^7,36^. We then compared the niche occupancy across the different regions, assessing the percentage of generalists and specialists microbes. In general, the proportion of generalists was higher than the specialists in all regions, revealing that the most proportion of microbial species can be found spread in every studied site. Interestingly, the proportion of generalists was higher between Mata and Sertão (37.7%), and the relative high share of generalists in these two regions could be related to the more chemical similarities between them, when compared to Agreste. In a study with species sorting, the authors found that a larger fraction of the variation in bacterial community composition could be explained by environmental factors in case of generalists^37^. On the other hand, our data showed that Agreste and Sertão present favorable conditions for specialists, showing a higher percentage of this group compared to Mata. This pattern can be associated with edaphic conditions found in Mata, such as higher rainfall and total organic C content, mainly in ‘Vitoria de Santo Antao’ (VSA), which may favor generalists bacteria^38^. On the other hand, Agreste and Sertão present stressful conditions to microbial life, evidenced by semiarid conditions, such as drought and higher temperatures in which may impose a strong selective effect on the bacterial community^39^. It has been shown that specialists are highly responsive to environmental disturbances, such as stresses and changes in soil chemical properties^40^. However, these authors also suggested that specialists are more susceptible to extinction than generalists when their habitat conditions are not favorable.

Finally, the network analysis was used to disentangle the dynamics of the microbial communities across the three distinct regions. Network analysis is a powerful approach to analyze the connections among microbial communities, and it is a possibility to infer information regarding soil nutrient cycling and health^41^. In addition, changes in microbial community structure also influence the microbial co-occurrence network patterns. Our results indicated that Sertão showed a more connected bacterial community, reflected by a higher number of nodes and edges, followed by Mata and Agreste. The enhanced network complexity in these areas can be a consequence of stronger bacterial interactions in the soil. Thus, the complex bacterial network in the Sertão can be more likely due to a combination of decreased diversity and nutrient contents. Interestingly, this area presented the lowest cowpea yield, revealing that the complexity and the number of microbe’s interactions do not reflect in higher plant productivity. Also, a more connected bacterial community in Sertão indicates the presence of different groups of nodes with high interconnections within, suggesting an increase in niche overlap and community stability, providing stronger resistance to extremophilic soil conditions but future studies are needed to confirm it^41^. Besides, different taxa have different roles within these networks, and keystone species could be crucial for ecosystem functioning and plant health^42^. The identification of the keystone species in Mata revealed that some groups are related to plant-growth promotion (PGP), such as *Bradyrhizobium* and Bacillales, which could explain the higher cowpea productivity in this region. Besides, some keystone species in Agreste were affiliated to bacterial groups also considered PGP, such as Rhizobiales and *Solibacillus.* Interestingly, the majority of the keystone species in the Sertão belong to the Actinobacteria phylum, the most abundant group found in this region. Together, our analysis showed the soil microbiome is shaped by different soil properties, and that microbiome composition and structure are linked to cowpea production. Interestingly, van der Heijden and Hartmann^42^ hypothesized that plant performance increases with increasing microbiome diversity, and our data showed higher cowpea yield in the areas with higher microbial diversity. Also, they suggested that plant performance depends on the presence of particular microbial species, i.e. the keystone species. Again, our analysis revealed that identified keystone species in the Mata, where the cowpea production was higher, were affiliated to already known microbial groups considered plant-growth promoting rhizobacteria.

## Conclusions

This study showed that the different soil conditions influence soil bacterial community and could be a predictor of the distinct performance of cowpea production. In general, our results have shown that the structure and composition of the bacterial community varied clearly between different regions (Mata, Agreste, and Sertão). Interestingly, the areas with higher cowpea yield presented higher microbial diversity along with keystone species related to plant-growth promotion, revealing that the soil microbiome has a fundamental role in plant productivity. Since cowpea is an important legume species cropped in the state of Pernambuco even in all Northeastern, Brazil, the knowledge of bacterial community distribution and its relationships with nutrient cycling can help farmers to increase inputs efficiency and, consequently, productivity, since the cowpea is cropped usually in poor soils^43^.

## Methods

### Study area

**Figure 6:**
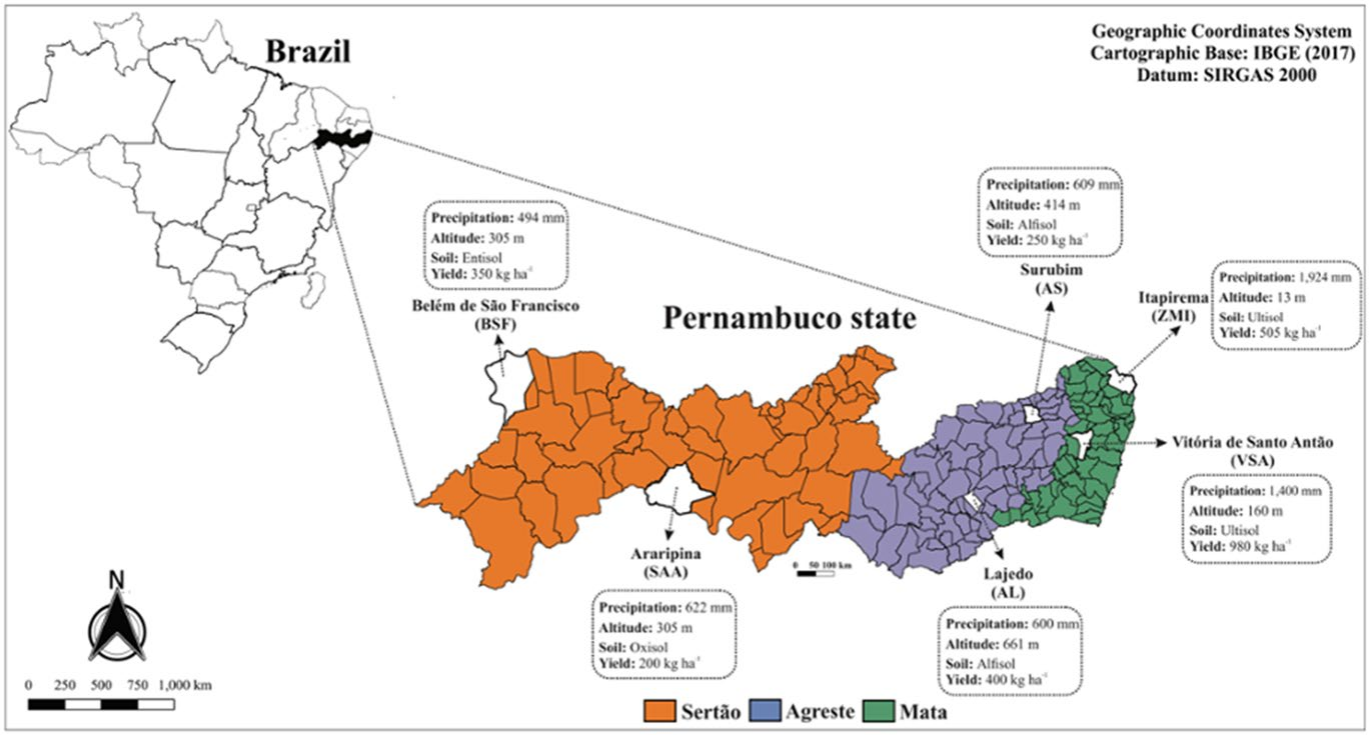
Geographic location of Pernambuco state, Brazil. Different colors mean the three studied ecoregions, i.e., Sertão, Agreste and Mata (including two localities in each). The map was created using QGIS software version 3.12.1 (https://qgis.osgeo.org).

The study was conducted in six localities belonging to three ecophysiographic regions found in the state of Pernambuco, North Eastern Brazil: Mata, Agreste, and Sertão. In this study, six localities were selected due to their differences on soil conditions, temperature, altitude, and cowpea yield^44^ (Table 1; Fig. 6): Vitória de Santo Antão (VSA—08° 06′ 50″ S and 35° 17′ 29″ W) and Itapirema (ZMI—07° 33′ 38″ S and 35° 17′ 29″ W) (Mata); Surubim (AS—07° 49′ 59″ S and 35° 45′ 17″ W) and Lajedo (AL—08° 39′ 49″ S and 36° 19′ 12″ W) (Agreste); Belém do São Francisco (BSF—08° 45′ 14″ S and 38° 57′ 57″ W) and Araripina (SAA—07° 34′ 34″ S and 40° 29′ 54″ W) (Sertão). In each location, the site (4000 m^2^) was divided into four transects with 20 m wide and 50 m long (replication) where soil samples were collected at 0–20 cm depth in March 2019. All soil samples were immediately stored in sealed plastic bags and transported in an icebox to the laboratory. A portion of the soil samples was stored in bags and kept at  − 20 °C for DNA analysis and another portion was air-dried, sieved through a 2-mm screen, and homogenized for chemical and physical analyses.

### Chemical and physical analysis

Soil chemical and physical properties were determined and measured using standard laboratory protocols^45^. Briefly, the soil pH was determined in a 1:2.5 soil/water extract, while that Al^3+^, Ca^2+^, and Mg^2+^ were estimated by the titrimetric method. Available P was estimated by colorimetry, and exchangeable Na^+^ and K^+^ were determined by photometry. Total organic C (TOC) was determined by the wet combustion method^46^. The percentage of sand, silt, and clay was estimated by the protocol described by the IBGE^44^. The comparison of soil attributes between the areas was performed using one-way ANOVA followed by the Tukey post hoc test (*p* < 0.05).

### DNA extraction, library preparation, and data processing

Soil samples (0.5 g) was used to extract DNA by using the DNA Isolation Kit (Power Soil MoBIO Laboratories, Carlsbad, CA, USA). The DNA extraction was performed in triplicate for each soil sample.

The amplicon library of the 16S rRNA gene V4 region was prepared as previously described (Illumina Team, 2013), using the region-specific primers (515F/806R)^47^. The first step of the amplification comprised 25 μL reaction containing the following: 14.8 μL of nuclease-free water (Certified Nuclease-free, Promega, Madison, WI, USA), 2.5 μL of 10X High Fidelity PCR Buffer (Invitrogen, Carlsbad, CA, USA), 1.0 μL of 50 mM MgSO_4_, 0.5 μL of each primer (10 μM concentration, 200 pM final concentration), 1.0 unit of Platinum Taq polymerase High Fidelity (Invitrogen, Carlsbad, CA, USA), and 4.0 μL of template DNA (10 ng). The conditions for PCR were as follows: 94 °C for 4 min to denature the DNA, with 25 cycles at 94 °C for 45 s, 60 °C for 60 s, and 72 °C for 2 min, with a final extension of 10 min at 72 °C. In the second step indexing PCR, a unique pair of Illumina Nextera XT indexes (Illumina, San Diego, CA) was added to both ends of the amplified products. Each 50 μL reaction contained the following: 23.5 μL of nuclease-free water (Certified Nuclease-free, Promega, Madison, WI, USA), 5.0 μL of 10X High Fidelity PCR Buffer (Invitrogen, Carlsbad, CA, USA), 4.8 μL of 25 mM MgSO_4_, 1.5 μL of dNTP (10 mM each), 5.0 μL of each Nextera XT index (Illumina, San Diego, CA, USA), 1.0 unit of Platinum Taq polymerase High Fidelity (Invitrogen, Carlsbad, CA, USA), and 5.0 μL of each product from previous PCR. The conditions for this second round PCR were as follows: 95 °C for 3 min to denature the DNA, with 8 cycles at 95 °C for 30 s, 55 °C for 30 s, and 72 °C for 30 min, with a final extension of 5 min at 72 °C.

PCR indexing clean up and quantification (Qubit 2.0 fluorometer) were performed according to Caporaso et al.^47^. Afterward, different volumes of each library were pooled into a single tube such that each amplicon was represented equally. The molarity of the pool was determined and diluted to 2 nM, denatured, and then diluted to a final concentration of 8.0 pM with a 20% PhiX (Illumina, San Diego, CA, USA) spike for loading into the Illumina MiSeq sequencing machine (Illumina, San Diego, CA, USA)^48^.

The 16S rRNA gene sequencing data were processed using QIIME 2 version 2019.10. Firstly, the paired-end sequences were merged using PEAR^49^, the sequences were demultiplexed and quality control was carried out using DADA2^50^, using the consensus method to remove any remaining chimeric and low-quality sequences. After filtering, approximately 2.15 million high-quality reads (on average, ~ 89,000 reads per sample) were obtained. Afterward, the samples were rarefied to 54,500 sequences, following the number of the lowest sample, and singletons and doubletons were removed. The taxonomic affiliation was performed at 97% of similarity using the Silva database v. 132^51^, and the generated matrix was further used for statistical analyses. The sequences are submitted to the NCBI Sequence Read Archive under the identification PRJNA646266.

### Data analysis

The statistical analyses were performed comparing the regions (i.e. the three distinct ecoregions) and the localities. Data were presented together when the effect of the sites was negligible. Initially, the data was checked for normal distribution using the Shapiro–Wilk test, and homogeneity of variance using Levene’s test, which indicated non-normal distribution. Thus, canonical correspondence analysis (CCA) was used to assess the bacterial community structure and correlate with soil parameters. Forward selection (FS) and the Monte Carlo permutation test were applied with 1000 random permutations to verify the significance of soil properties upon the microbial community. The CCA plot was generated using Canoco 5 (Biometrics, Wageningen, The Netherlands)^52^. We used two-way PERMANOVA^53^ to test if region and site harbored significantly different microbial communities. Measurements of Shannon’s diversity and richness were calculated based on the taxonomic matrix at the OTU level using PAST 4.01 software^54^.

To compare the composition of the bacterial communities between regions we used the Statistical Analysis of Metagenomic Profile (STAMP) software^55^. For this, the OTU table at the phylum and family level generated from QIIME 2 was used as input. P-values were calculated using a two-sided Tukey–Kramer test, and the correction was made using the Benjamini–Hochberg false discovery rate^56^. For visualization, a heatmap was constructed based on z-score transformed phylum abundance to improve normality and homogeneity of the variances, using the ‘pheatmap’ package in R (R Development Core Team)^57^. The color key relates the heatmap colors to the standard score (z-score), i.e., the deviation from row mean in units of standard deviations above or below the mean.

To explore the relationship between the relative abundance of microbial groups and soil properties, we calculated Spearman’s rank correlation coefficients using the ‘multtest’ package in R, and the correction was made using Benjamini–Hochberg FDR. For visualization, a heatmap was construct using the ‘corrplot’ package in R, where significant (*P* < 0.05) positive and negative correlations are represented in blue and red, respectively.

In addition, the bacterial community dynamics was assessed comparing the three regions. First, the niche occupancy, i.e. the percentage of generalists and specialists in each region, was verified by the multinomial species classification method using the ‘vegan’ package and the function ‘clamtest’ in R, with individual test alpha of 0.05 and coverage limit of 10. This method compares the abundance of the microbial communities between two environments and classifies the microbes into different classes, namely specialists, generalists, and too rare^58,59^. Them to assess the complexity of the communities’ interactions we performed network analysis. For this, SparCC correlations^60^ were calculated, and only significant (*p* < 0.01) and strong (> 0.9 or <  − 0.9) correlations were selected. For network visualization and the calculation of topological properties, we used the interactive platform Gephi^61^.

## Supplementary Information

Supplementary information.
