# Supplementary material for: Distinct bacterial community structure and composition along different cowpea producing ecoregions in Northeastern Brazil
Source: Sci Rep. 2021 Jan 12;11:831. doi: 10.1038/s41598-020-80840-x (PMC7804402; doi:10.1038/s41598-020-80840-x)
Supplement: Supplementary file 1 — Supplementary information. [file 41598_2020_80840_MOESM1_ESM.docx]

**Distinct bacterial community structure and composition along different cowpea producing ecoregions in Northeastern Brazil**

Luciana de Sousa Lopes^1^, Lucas William Mendes^2^, Jadson Emanuel Lopes Antunes^1^, Louise Melo de Souza Oliveira^1^, Vania Maria Maciel Melo^3^, Arthur Prudêncio de Araujo Pereira^4^, Antonio Félix da Costa^5^, José de Paula Oliveira^5^, Cosme Rafael Martinez^6^, Marcia do Vale Barreto Figueiredo^5^, Ademir Sérgio Ferreira Araujo^1^*

**Supplementary Table 1.** OTUs with more betweeness centrality and number of correlations for each treatment.

| **Treatment** | **OTU** | **Phylum** | **Lowest classification** | **Betweeness Centrality^a^** | **Degree^b^** |
| --- | --- | --- | --- | --- | --- |
| **Mata** | OTU 490 | Chloroflexi | Anaerolineae | 1443.72 | 31 |
|  | OTU 518 | Chloroflexi | Roseiflexacae | 1347.40 | 114 |
|  | OTU 773 | Firmicutes | Bacillales | 1283.68 | 99 |
|  | OTU 1135 | Proteobacteria | *Bradyrhizobium* | 1110.48 | 96 |
|  | OTU 1321 | Proteobacteria | Deltaproteobacteria | 916.79 | 19 |
| **Sertão** | OTU 303 | Actinobacteria | Solirubrobacterales | 2478.90 | 187 |
|  | OTU 249 | Actinobacteria | *Nonomuraea* | 2330.65 | 25 |
|  | OTU 310 | Actinobacteria | *Solirubrobacter* | 2229.49 | 190 |
|  | OTU 118 | Acidobacteria | Subgroup 6 | 2072.52 | 10 |
|  | OTU 283 | Actinobacteria | *Rubrobacter* | 1947.72 | 209 |
| **Agreste** | OTU 223 | Actinobacteria | *Nocardioides* | 3229.90 | 46 |
|  | OTU 1149 | Proteobacteria | Rhizobiales | 2876.12 | 12 |
|  | OTU 863 | Gemmatimonadetes | Unclassified | 2814.73 | 12 |
|  | OTU 1369 | Proteobacteria | Nitrosomonadaceae | 2678.01 | 56 |
|  | OTU 755 | Firmicutes | *Solibacillus* | 2320.43 | 9 |

^a^The fraction of cases in which a node lies on the shortest path between all pair of other nodes, interpreted as keystone species.

^b^Number of connections/correlations obtained by SparCC analysis;
